# Identification of the viral RNA promoter stem loop A (SLA)-binding site on Zika virus polymerase NS5

**DOI:** 10.1038/s41598-020-70094-y

**Published:** 2020-08-06

**Authors:** Paul J. Bujalowski, Wlodzimierz Bujalowski, Kyung H. Choi

**Affiliations:** Department of Biochemistry and Molecular Biology, Sealy Center for Structural Biology and Molecular Biophysics, The University of Texas Medical Branch, Galveston, TX 77555 USA

**Keywords:** RNA-binding proteins, Dengue virus, Kinetics

## Abstract

Zika virus has recently emerged as an important human pathogen that has spread to more than 60 countries. Infection of a pregnant woman with Zika virus can cause severe brain malformations in the child such as microcephaly and other birth defects. Despite the medical importance of Zika virus infection, the mechanism of viral replication, a process commonly targeted by antiviral therapeutics, is not well understood. Stem-loop A (SLA), located in the 5′ untranslated region of the viral genome, acts as a promotor for viral replication and thus is critical for recognition of the viral genome by the viral polymerase NS5. However, how NS5 engages SLA is not clear. We have quantitatively examined the intrinsic affinities between Zika virus SLA and NS5, and identified the SLA-binding site on NS5. Amino acid substitutions in the thumb subdomain of the RNA-dependent RNA polymerase (RdRp) and the methyltransferase (MTase) domain reduced SLA-binding affinity, indicating that they each are part of the SLA-binding site. Furthermore, stopped-flow kinetic analysis of Zika NS5-, RdRp- and MTase–SLA interactions identified distinct intermediates during NS5 and SLA complex formation. These data suggest a model for SLA recognition and the initiation of flaviviral replication by NS5.

## Introduction

Zika virus (ZIKV) is a member of the family *Flaviviridae*, and since 2007 has emerged as a major threat to global health, causing a series of epidemics in Micronesia, the South Pacific and both Americas^1^. Currently, the virus has been reported in 65 countries^2,3^. ZIKV is closely related to human pathogens such as tick-borne encephalitis virus, dengue virus (DENV), West Nile virus, yellow fever virus, and Japanese encephalitis virus^4,5^. ZIKV can be transmitted sexually and through blood transfusions, and spread from a pregnant woman to her fetus. ZIKV infection of a pregnant woman can cause severe brain malformations in her child including microcephaly and other birth defects^6,7^. Moreover, in adults, ZIKV has been linked to Guillain–Barré syndrome, a condition wherein a person’s immune system attacks their peripheral nerves^8,9^. In severe cases, this can lead to near-total paralysis and death.

The structure of the ZIKV genome is similar to other flaviviral genomes. The 11 kb long positive sense RNA contains an open reading frame (ORF) as well as 5′ and 3′ untranslated regions (UTRs) that regulate viral replication^10–12^. The ORF is translated into a polyprotein, C-prM-E-NS1-NS2A-NS2B-NS3-NS4A-NS4B-NS5, which is subsequently cleaved into three structural (C, prM, and E) and seven non-structural (NS) proteins. The viral NS proteins, along with viral RNA and unidentified host proteins, self-assemble on the cytoplasmic side of the ER membrane to form a viral replication complex that carries out genome replication. Although all NS proteins are required to form a viral replication complex, only NS3 and NS5 enzyme activities are involved in viral genome replication. NS3 consists of an N-terminal serine protease domain, which requires NS2B as a cofactor, and a C-terminal helicase domain. The helicase domain also has nucleoside-triphosphatase (NTPase) activity and 5′-RNA triphosphatase activity, which hydrolyzes the γ-phosphate of RNA for RNA capping^13^. NS5 is the viral replicative polymerase responsible for genome synthesis^4,14,15^. Full-length NS5 encompasses approximately 900 amino acids and is composed of two functionally different domains^16–21^. The smaller N-terminal domain functions as a methyltransferase (MTase), whose activity is necessary for the 5′-RNA cap formation and methylation that are important for viral RNA recognition by the host cell translational apparatus^22,23^. The larger C-terminal domain possesses RNA-dependent RNA polymerase (RdRp) activity, containing the active site for RNA synthesis^24^.

**Figure 1:**
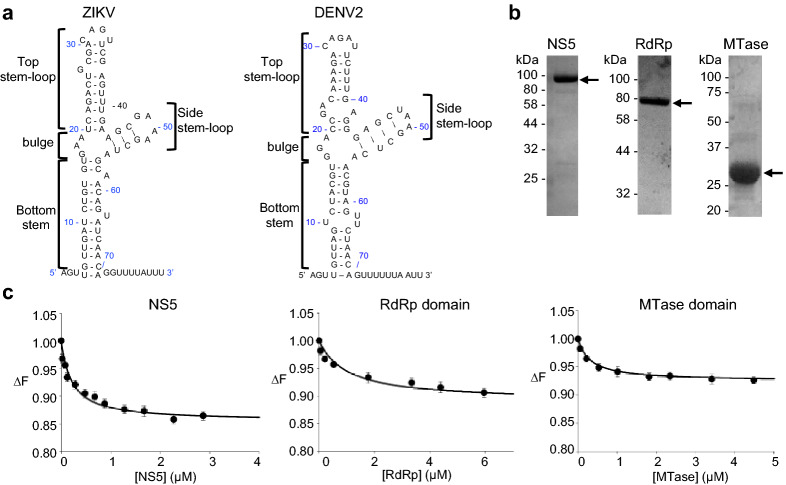
Full-length Zika NS5, the RdRp domain, and the MTase domain interact with SLA. (**a**) The predicted secondary structures of the Zika and dengue virus SLA. The secondary structure of the first 80 nt of ZIKV (KU527068.1) used in this study was predicted by mFold^42^. The predicted secondary structure of DENV2 SLA (NC_001474) is shown on the right. Both SLAs consists of the top stem-loop, side stem-loop, and bottom stem with a bulge between the top and bottom stem. Sequence identity between ZIKV and DENV2 SLA is 64%. (**b**) SDS-PAGE analysis of full-length NS5, RdRp and MTase. The positions of protein molecular weight markers are shown on the left. The expected sizes of the NS5, RdRp, and MTase are 100, 72, and 30 kDa, respectively. (**c**) Fluorescence titrations of fluorescein-labeled ZIKV SLA with NS5, the RdRp domain, and the MTase domain. The binding reactions were carried out with 15 nM fluorescein-labeled SLA in buffer B1 (50 mM Tris, pH 8.0, 150 mM NaCl, 1 mM MgCl_2_, 2 mM β-mercaptoethanol and 10% glycerol) at 20 °C with λ_ex_ = 480 nm, λ_em_ = 520 nm. The solid lines are nonlinear least squares fits of the titration curves (Eq. 11) with K_NS5_ = 3.5 × 10^6^ M^−1^ (*K*_*d*_= 0.28 μM, R^2^ = 0.98), K_RdRp_ = 1.9 × 10^6^ M^−1^ (*K*_*d*_= 0.53 μM, R^2^ = 0.97), and K_MTase_ = 1.7 × 10^6^ M^−1^ (*K*_*d*_= 0.59 μM, R^2^ = 0.99). Each experiment was carried out in triplicate. Error bars represent the standard deviations.

In flaviviruses, the 5′-UTR of the viral genome includes a structured stem-loop, called stem-loop A (SLA, nt 1- 70) (Fig. 1a), which functions as a promoter and recruits the viral polymerase NS5 to initiate RNA synthesis at the 3′-end of the genome^10–12,25,26^. Although NS5 can synthesize RNA from short RNA templates, NS5 cannot synthesize RNA from a sub-genomic RNA template that lacks SLA^11^. A predicted ‘Y’-shaped secondary structure of SLA is conserved in all dengue virus serotypes (DENV1-4) as well as in other flaviviruses such as ZIKV and West Nile virus^12,27,28^, suggesting that flaviviruses share a common promoter structure and initiation mechanism. Additionally, SLA is required for 5′ cap methylation at the N7 position, indicating that the MTase domain of NS5 recognizes structural features of the SLA to carry out the methylation reaction^29^. Despite the importance of SLA in initiating both of these critical aspects of viral replication (RNA synthesis and RNA capping), the exact roles each of the NS5 domains play in recognizing and binding SLA are not well understood. In particular, the SLA-binding site on NS5 has not been identified. Thus, it is not clear whether both NS5 domains are involved in SLA binding or whether one of the two domains regulates promotor binding.

To elucidate the mechanism of the first step of flavivirus replication, i.e., the NS5-RNA genome recognition process, we have previously examined DENV NS5 interactions with SLA, ssRNA, and dsRNA using fluorescence titration techniques^30,31^. DENV NS5 binds SLA specifically with 83 nM affinity and a 1:1 stoichiometry^30^. Here, we have examined the binding affinities of ZIKV SLA with the full-length NS5 and its two individual RdRp and MTase domains, and identified the location of the SLA-binding site on the surface of NS5. Our data show that SLA interacts with specific positively charged surface located on both the RdRp and MTase domains of NS5. We further determined the kinetics of Zika NS5-, RdRp- and MTase–SLA complex formation, and observed the presence of intermediates in the NS5–SLA complex reaction.

## Results

### Full-length Zika NS5 and the individual RdRp and MTase domains interact with Zika SLA

Flavivirus replication is initiated when the viral polymerase NS5 identifies the viral genome via specific recognition of the SLA^10–12^. Accordingly, flavivirus NS5 has been shown to bind SLA with high affinity^10,30,32^. We have previously determined that DENV NS5 and SLA interact with 83 nM affinity using fluorescence measurements^30^. However, the roles each of the NS5 domains play in recognizing and binding SLA are not well understood. Here, we used ZIKV NS5 and the individual RdRp and MTase domains, and examined their binding affinities using the ZIKV SLA (80 nt, Fig. 1a) fluorescein-labeled at the 3′ end (SLA-F). Sequence identities between DENV and ZIKV NS5 and SLA are 67% and 64%, respectively. Fluorescence titrations of SLA-F with ZIKV NS5, RdRp domain and MTase domain (Fig. 1b) were carried out in buffer B1 (50 mM Tris adjusted to pH 8.0 with HCl at 20 °C, 150 mM NaCl, 1 mM MgCl_2_, 2 mM β-mercaptoethanol, and 10% glycerol). The formation of the complex between the ZIKV NS5 proteins and SLA-F causes a decrease in nucleic acid fluorescence (Fig. 1c). The maximum observed value of fluorescence quenching ranged from 15% for the full-length NS5, 9% for the RdRp domain, to 7% for the MTase domain. The solid lines in Fig. 1c are the nonlinear least-squares fits of Eq. (11) to the experimental data (see “Methods” section). The binding constants are listed in Table 1. The full-length ZIKV NS5 binds SLA with a binding constant of K_NS5_ = 3.5 × 10^6^ M^−1^ (*K*_*d*_ = 0.28 μM). The binding constants for the RdRp domain and MTase domain are K_RdRp_ = 1.9 × 10^6^ M^−1^ (*K*_*d*_ = 0.53 μM) and K_MTase_ = 1.7 × 10^6^ M^−1^ (*K*_*d*_ = 0.59 μM), respectively. Thus, both MTase and RdRp domains individually interact with SLA-F with  approximately  two-fold lower affinities than the full-length NS5.

**Table 1:** Effects of Zika NS5 mutations on SLA-binding affinity.

Protein	Location of mutations	Binding constant, K (10^6^ M^−1^)^a^	Dissociation constant, *Kd* (μM)	Fold change
WT NS5		3.5 ± 0.6	0.28	–
MTase domain		1.7 ± 0.3	0.59	2.1
RdRp domain		1.9 ± 0.3	0.53	1.8
NS5 mutant				
K388A-K390A-R391A	Palm	3.5 ± 0.6	0.28	1.0
R640A-R641A	Fingers	3.5 ± 0.6	0.28	1.0
R775A	Thumb	3.5 ± 0.6	0.28	1.0
R771A-R772A-R775A	Thumb	2.0 ± 0.3	0.50	1.8
R771A-R772A-R775A-K843A-R844A	Thumb	1.4 ± 0.2	0.71	2.5
K843A-R844A	Thumb	3.5 ± 0.6	0.28	1.0
R858A	Thumb	3.5 ± 0.6	0.28	1.0
R891A	Thumb/C-terminus	3.5 ± 0.6	0.28	1.0

^a^All binding constants were measured in Buffer B1 (50 mM Tris–HCl, pH 8.0, 150 mM NaCl, 1 mM MgCl_2_, 2 mM β-mercaptoethanol, and 10% glycerol).

### The thumb subdomain of the RdRp domain is responsible for binding to SLA

**Figure 2:**
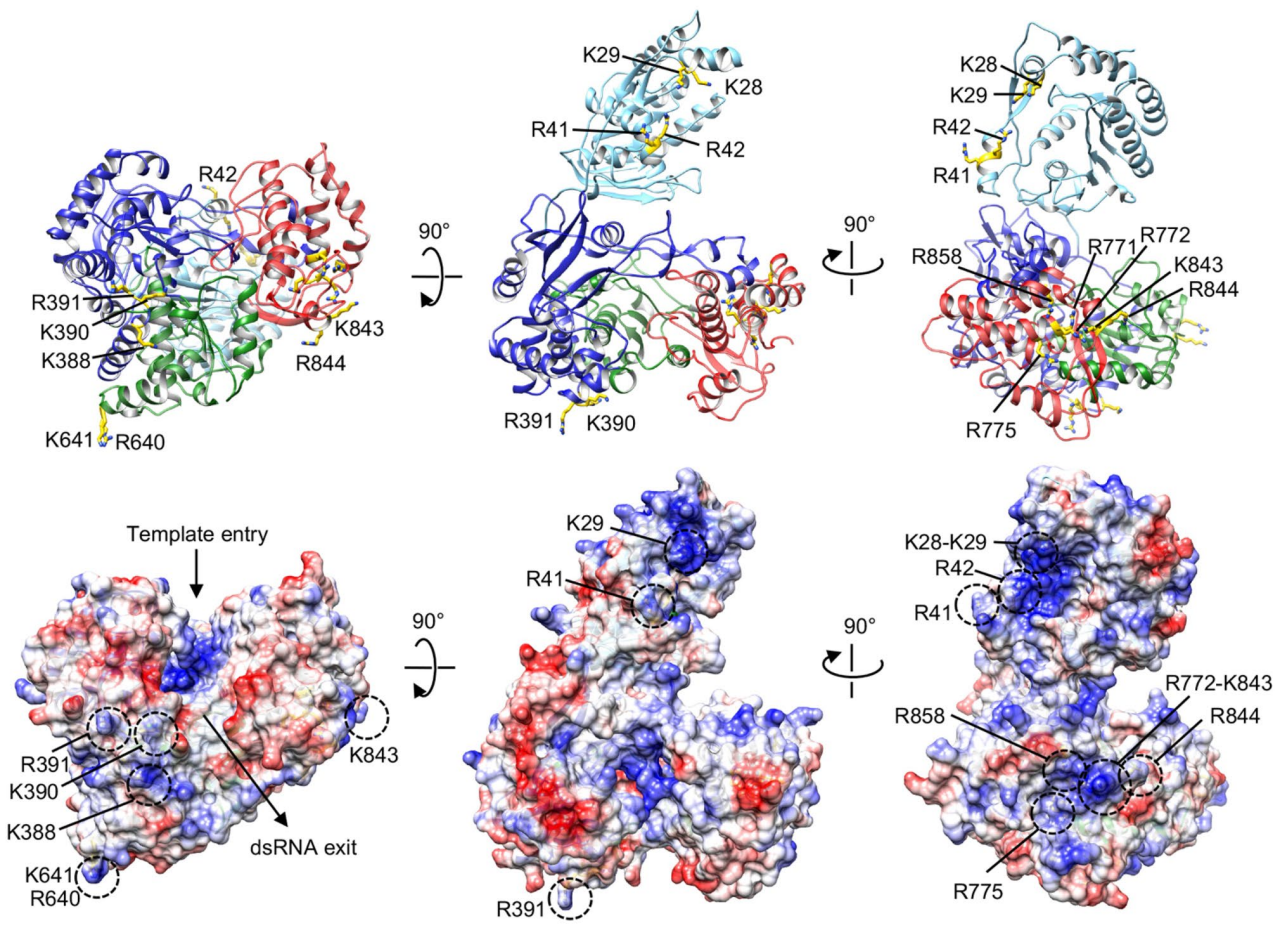
Mapping of Zika NS5 residues potentially involved in the recognition of SLA. Ribbon diagrams (top) and electrostatic surfaces (bottom) of Zika NS5 (PDB accession code 5U0B) are shown in three orientations with color-coded subdomains of the RdRp domain (thumb, red; palm, green; fingers, blue) and the MTase domain (cyan). Residues selected for mutagenesis are labeled and colored in yellow in ribbon diagram.

**Figure 3:**
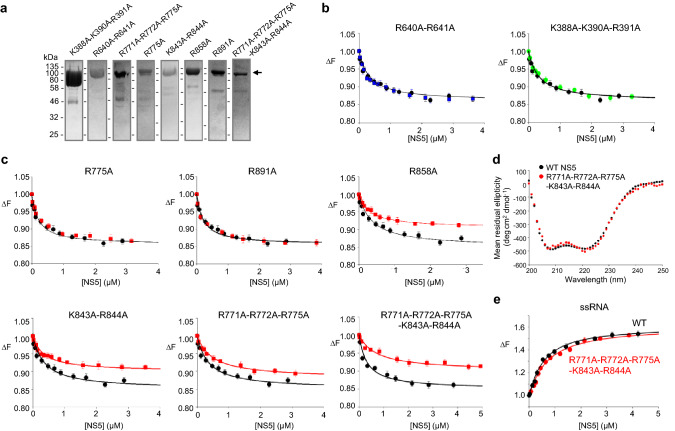
Mutations in the thumb subdomain decrease NS5 and SLA interactions. (**a**) SDS-PAGE of purified ZIKV NS5 mutants. Positions of molecular weight markers are shown on the left. (**b**) Fluorescence titrations of the fingers and palm subdomain mutants with SLA. NS5 mutants R640A-R641A (fingers subdomain, blue) and K388A-K390A-R391A (palm subdomain, green) interactions with fluorescein-labeled SLA in buffer B1 were measured by the fluorescence change at 20 °C. Titration of NS5 wild-type is shown in black. The solid lines are nonlinear least squares fits of the titration curves to Eq. (11) for the wild-type and mutants. Both R640A-R641A and K388A-K390A-R391A bind SLA with wild-type binding affinity of K = 3.5 × 10^6^ M^−1^ (*K*_*d*_= 0.28 μM, R^2^ = 0.98 and R^2^ = 0.99 respectively). Each experiment was carried out in triplicate. Error bars represent the standard deviations. (**c**) Fluorescence titrations of the thumb subdomain mutants with SLA. The titration curves were fit to Eq. (11) using nonlinear least squares fits. The R775A and R891A mutants show identical SLA binding curves to wild-type NS5 (R^2^ = 0.98 and R^2^ = 0.99, respectively). The R858A and K843A-R844A mutants bind SLA with wild-type affinity, but show a reduced maximal value of the fluorescence signal (R^2^ = 0.97 and R^2^ = 0.98, respectively). The R771A-R772A-R775A and R771A-R772A-R775A-K843A-R844A bind Zika SLA with reduced affinities of K = 2.0 × 10^6^ (*K*_*d*_= 0.50 μM, R^2^ = 0.98) and 1.4 × 10^6^ M^−1^ (*K*_*d*_= 0.71 μM, R^2^ = 0.98), respectively. Each experiment was carried out in triplicate. Error bars represent the standard deviations. (**d**) CD spectra of wild-type NS5 and mutant R771A-R772A-R775A-K843A-R844A. The CD data indicate that there is no structural change in the NS5 mutant. (**e**) Interactions of Zika NS5 proteins with 20-mer ssRNA. Fluorescence changes of the wild type (black) and mutant NS5 (R771A-R772A-R775A-K843A-R844A, red) upon binding εA(pεA)_19_ (λ_ex_ = 325 nm; λ_em_ = 410 nm) were measured in buffer B1. The solid lines are nonlinear least squares fits of the titration curve (Eq. 11) with K_wt_ = 2.0 × 10^6^ (*K*_*d*_= 0.50 μM, R^2^ = 0.98) and K_mut_ = 1.5 × 10^6^ M^−1^ (*K*_*d*_= 0.67 μM, R^2^ = 0.98). Each experiment was carried out in triplicate. Error bars represent the standard deviations.

Because both RdRp and MTase domains are capable of independently binding to SLA with similar affinities, the SLA-binding site on NS5 likely involves both the RdRp and MTase domains. To identify the SLA-binding site in NS5, we calculated the electrostatic surface of the ZIKV NS5 structure^19^ to look for positively charged patches that would be complementary to the negatively charged SLA (Fig. 2). The RdRp domain is composed of three subdomains, fingers, palm and thumb subdomains, and positively charged residues were selected from all three subdomains for substitutions in NS5. Figure 2 shows the locations of targeted residues within NS5. The selected NS5 mutants were generated by site-directed mutagenesis and purified similar to the wild-type protein (Fig. 3a). The binding constants for NS5 mutants were then measured using SLA-F in buffer B1 (Fig. 3b–c). The binding constants are listed in Table 1. The binding assay showed that mutations in the fingers and palm subdomains did not affect SLA binding (Fig. 3b). For example, a double mutant within the fingers subdomain (R640A-R641A) and a triple mutant in the palm subdomain (K388A-K390A-R391A) bind to SLA with indistinguishable affinities compared to the wide-type NS5. Thus, these regions in the fingers and palm subdomains are not directly involved in interactions with SLA. The residues K388, K390, and R391 in the palm subdomain are located near the product exit site of the RdRp domain likely involved in the dsRNA product binding (Fig. 2). Mutations of K387A-K388A or K388A-K389A in DENV4 NS5 (corresponding to K388, H389 and K390 in ZIKV NS5) have shown to reduce virus titers by 1.4 – 2.4 log_10_ PFU/ml^33^. Our result thus suggests that the dsRNA product binding site does not overlap with the SLA-binding site.

In contrast, several mutants in the thumb subdomain showed reduced binding affinities and/or altered the magnitude of the fluorescence signal (Fig. 3c). The thumb subdomain contains a large area of positively charged surface that include R771, R772, R775, K843, and R844 (Fig. 2). Thus, we generated six mutations in the area (Fig. 3a,c). The triple mutant, R771A-R772A-R775A showed the binding affinity of 2.0 × 10^6^ M^−1^ (*K*_*d*_ = 0.50 μM), a 1.8-fold reduction from the wild-type affinity (Fig. 3c and Table 1). Since K843 and R844 are located in the positively charged patch near the mutated R771, R772, and R775, we tested if additional mutations within the patch reduce the binding to SLA even further. NS5 mutant containing R771A, R772A, R775A, K843A, and R844A indeed displayed a weaker affinity to SLA with K = 1.4 × 10^6^ M^−1^ (*K*_*d*_ = 0.71 μM) (Fig. 3c and Table 1). To determine if the reduced affinity of the R771A-R772A-R775A-K843A-R844A mutant is caused by the structural changes of the mutant, we compared the CD spectra for wild-type NS5 and the mutant (Fig. 3d). The CD spectra were similar, indicating that the secondary structure of NS5 has not been affected by mutations. Thus, the reduced affinity of the R771A-R772A-R775A-K843A-R844A mutant is caused by the loss of its direct interaction with SLA. We also generated single and double mutations in the region, but they did not significantly affect SLA binding, suggesting that other positively charged residues within the patch may compensate for the loss of their positively charged side chains (Fig. 2). For example, R775A shows the same binding affinity as the wild-type NS5 (Fig. 3c and Table 1). A single mutant of R858A showed decreased magnitude of the fluorescence signal, although the binding affinity remained the same as for wild-type NS5 (Fig. 3c and Table 1). This indicates that Ala substitution in R858A affects the conformation of the bound SLA. A double mutant K843A-R844A also showed a decreased magnitude of the fluorescence signal suggesting that K843 and R844 also induce changes in the nucleic acid structure, although the mutations alone were not able to significantly reduce SLA binding (Fig. 3c and Table 1). We also tested if the C-terminal region of NS5 is involved in interactions with SLA. A single mutation of R888 to Ala in DENV NS5 was shown to produce a completely non-viable virus^34^. In the crystal structure of DENV NS5, R888 in the C-terminal helix is located near R772 of the positively charged patch^16^, suggesting that R888 could form a part of the SLA-binding surface. The corresponding residue R891 in ZIKV NS5 is disordered in the crystal structure^19^. However, the R891A mutant in ZIKV NS5 binds SLA with the wild-type affinity, suggesting that R891 at the C-terminal end of NS5 is not involved in the interactions with SLA (Fig. 3c and Table 1).

To determine if the SLA-binding site on the thumb subdomain is specific for SLA interaction, we next measured NS5 protein interactions with a nonspecific single-stranded RNA (ssRNA). Because the identified SLA-binding site does not overlap with the template-binding site (where ssRNA binds, Fig. 2), the SLA-binding site mutant should be able to bind nonspecific ssRNAs. We thus determined the binding affinity of NS5 proteins (wild-type and the R771A-R772A-R775A-K843A-R844A mutant) with etheno-labeled A(pA)_19_, εA(pεA)_19_ (Fig. 3e). Wild-type and mutant NS5 bind εA(pεA)_19_ with a similar binding affinity of K_wt_ = 2.0 × 10^6^ M^−1^ (*K*_*d*_ = 0.50 μM) and K_mut_ = 1.5 × 10^6^ M^−1^ (*K*_*d*_ = 0.67 μM). Thus, the mutations that affect SLA binding in NS5 do not significantly change nonspecific ssRNA interaction, indicating that the positively charged patch in the thumb subdomain (R771, R772, R775, K843, and R844) specifically interacts with SLA.

### Mapping the SLA-binding region within the MTase domain

**Figure 4:**
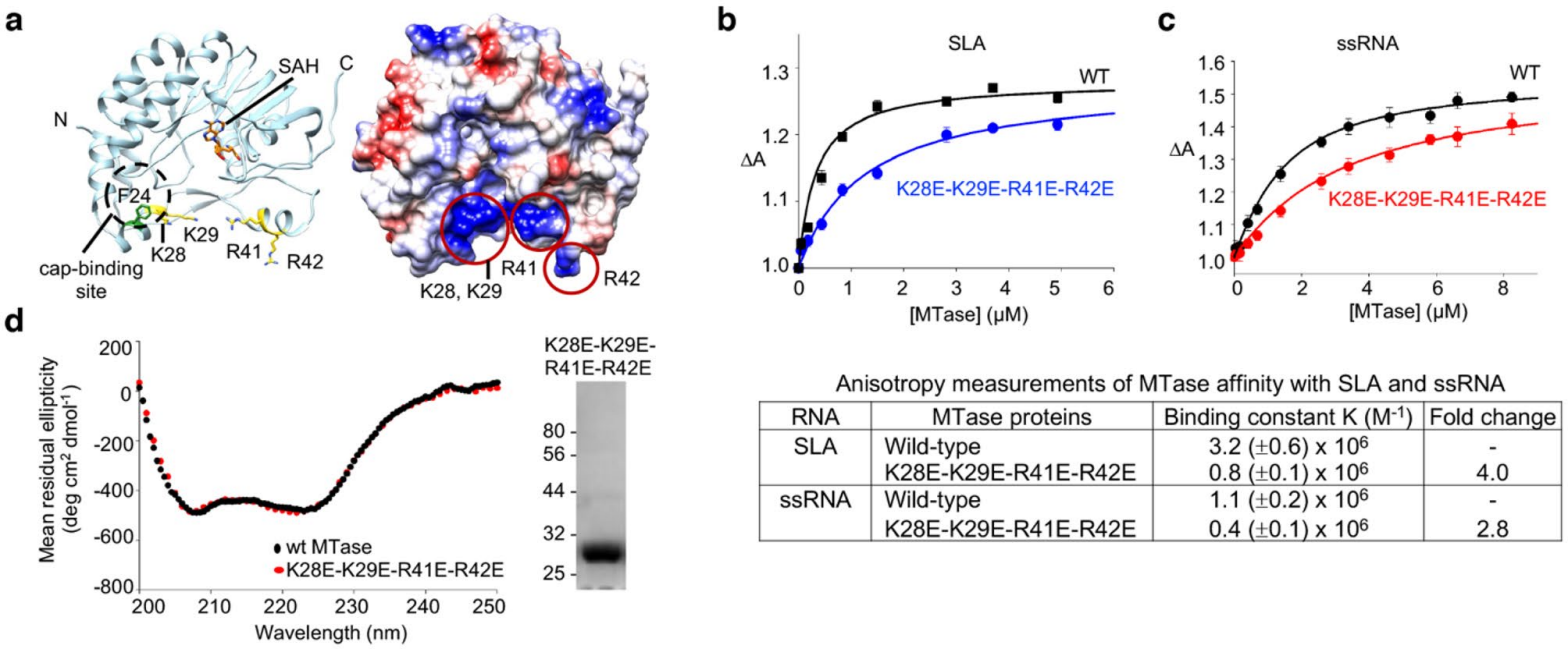
Mutations of the MTase domain affect SLA interactions. (**a**) ZIKV MTase structure. The locations of the mutated residues in the ZIKV MTase domain (PDB accession code 5U0B) are shown in a ribbon diagram (left) and an electrostatic surface (right). The coproduct S-adenosyl homocysteine (SAH) and the cap-binding site F24 are also indicated in the ribbon diagram. (**b**) Interactions of Zika SLA with MTase proteins. MTase wild-type (WT, black) and K28E-K29E-R41E-R42E (blue) interactions with fluorescein-labeled SLA in buffer B2 were analyzed by measuring the relative increase of fluorescence anisotropy of the labeled nucleic acid. The solid lines are nonlinear least squares fits of the titration curves with K_wt_ = 3.2 × 10^6^ (*K*_*d*_= 0.31 μM, R^2^ = 0.97) and K_mut_ = 0.8 × 10^6^ M^−1^ (*K*_*d*_= 1.25 μM, R^2^ = 0.98). The binding constants and relative binding affinities of MTase proteins are listed below. (**c**) Interactions of 20-mer ssRNA with Zika MTase proteins. MTase WT (black) and K28E-K29E-R41E-R42E (red) interactions with εA(pεA)_19_ were measured by fluorescence anisotropy (λ_ex_ = 325 nm, λ_em_ = 410 nm). The solid lines are nonlinear least squares fits of the titration curves with K_wt_ = 1.1 × 10^6^ (*K*_*d*_= 0.91 μM, R^2^ = 0.98) and K_mut_ = 0.4 × 10^6^ M^−1^ (*K*_*d*_= 2.5 μM, R^2^ = 0.98). The binding constants and relative binding affinities of MTase proteins are listed below. Each experiment (**b**,**c**) was measured in triplicate. Error bars represent the standard deviations. (**d**) Comparison of CD spectra between the wild-type MTase and the mutant K28E-K29E-R41E-R42E. The CD spectrum of the mutant MTase is identical to that of the wild-type MTase. The purity of the mutant MTase is analyzed by SDS-PAGE (right).

Our binding data show that the MTase domain alone interacts with SLA (Fig. 1c). Crystal structures of flavivirus MTases co-crystallized with GTP, cap analog GpppA, cofactor S-adenosyl-L-methione (SAM) or its product S-adenosyl-L-homocysteine (SAH) identified the catalytic site and cofactor binding sites^23,35^. The structures also suggest a putative RNA-binding site, consisting of K28, K29, R37, R41, R42, R57, and R213 (Fig. 4a). Several of these residues (K28, K29, R57, and R213) in DENV MTase have previously been mutated to Ala and their interactions with a short RNA examined^36^. The reduction in RNA binding varied by one to five-fold, with greatest effect observed for the K29A mutation. This putative RNA-binding site with a positively charged patch is located on the same surface as the positively charged patch in the RdRp domain (Fig. 2, right). To determine whether these MTase residues play a role in the NS5–SLA complex formation, we introduced a four-residue mutation, K28E-K29E-R41E-R42E, in the MTase domain construct and measured its interaction with SLA-F by fluorescence. However, the change in fluorescence signal for the MTase mutant was too small to accurately determine the binding constant. To increase the resolution of the analysis, we utilized the anisotropy signal to examine MTase and SLA interactions (see “Methods” section). Anisotropy titrations of SLA-F with wild-type and the mutant (K28E-K29E-R41E-R42E) MTase domain in buffer B2 (50 mM Tris at pH 8.0, 100 mM NaCl, 1 mM MgCl_2_, 2 mM β-mercaptoethanol, and 10% glycerol) at 20 °C are shown in Fig. 4b. The wild-type MTase domain binds SLA with a binding affinity K_wt_ = 3.2 × 10^6^ M^−1^ (*K*_*d*_ = 0.31 μM). This value measured by anisotropy is similar to the binding constant determined by the fluorescence titration in the same buffer B2, 2.5 × 10^6^ M^−1^ (*K*_*d*_ = 0.40 μM) (Fig. S1). The affinity of the mutant MTase K28E-K29E-R41E-R42E measured by anisotropy was K_mut_ = 0.8 × 10^6^ M^−1^ (*K*_*d*_ = 1.25 μM), i.e., it decreased fourfold compared to the wild-type MTase (Fig. 4b). The reduced SLA-binding affinity of the mutant was not caused by structural changes induced by the mutations, since the comparison of the CD spectra between the wild-type and mutant MTase show identical curves (Fig. 4d). These results indicate that the mutated residues are most likely involved in NS5–SLA interaction.

To determine if the decreased affinity is specific for SLA compared to non-specific ssRNA, the associations of the wild-type and mutant MTase were measured by fluorescence anisotropy with the fluorescently labeled ssRNA, εA(pεA)_19_ in buffer B2 (Fig. 4c). The wild-type MTase binds the ssRNA with K_wt_ = 1.1 × 10^6^ M^−1^ (*K*_*d*_ = 0.91 μM). The binding constant of the mutant MTase K28E-K29E-R41E-R42E for the ssRNA is K_mut_ = 0.4 × 10^6^ M^−1^ (*K*_*d*_ = 2.5 μM), a 2.8-fold reduction compared to the wild-type. The SLA-binding site identified in the MTase (K28, K29, R41, R42) partially overlaps with the capped RNA-binding site (K14, L17, N18, L20, F25, K29) determined by the crystal structures of MTase in complex with capped RNA^35,37^. Thus, it is likely that the SLA-binding site mutant in the MTase domain also reduces the ssRNA interaction. Nevertheless, the mutant MTase (K28E-K29E-R41E-R42E) reduced the SLA binding affinity more significantly than the ssRNA binding affinity (four-fold vs. 2.8-fold), indicating that the positively charged patch containing K28, K29, R41, and R42 is involved in SLA binding.

### Stopped-flow kinetics of ZIKV NS5, RdRp, and MTase binding to SLA

**Figure 5:**
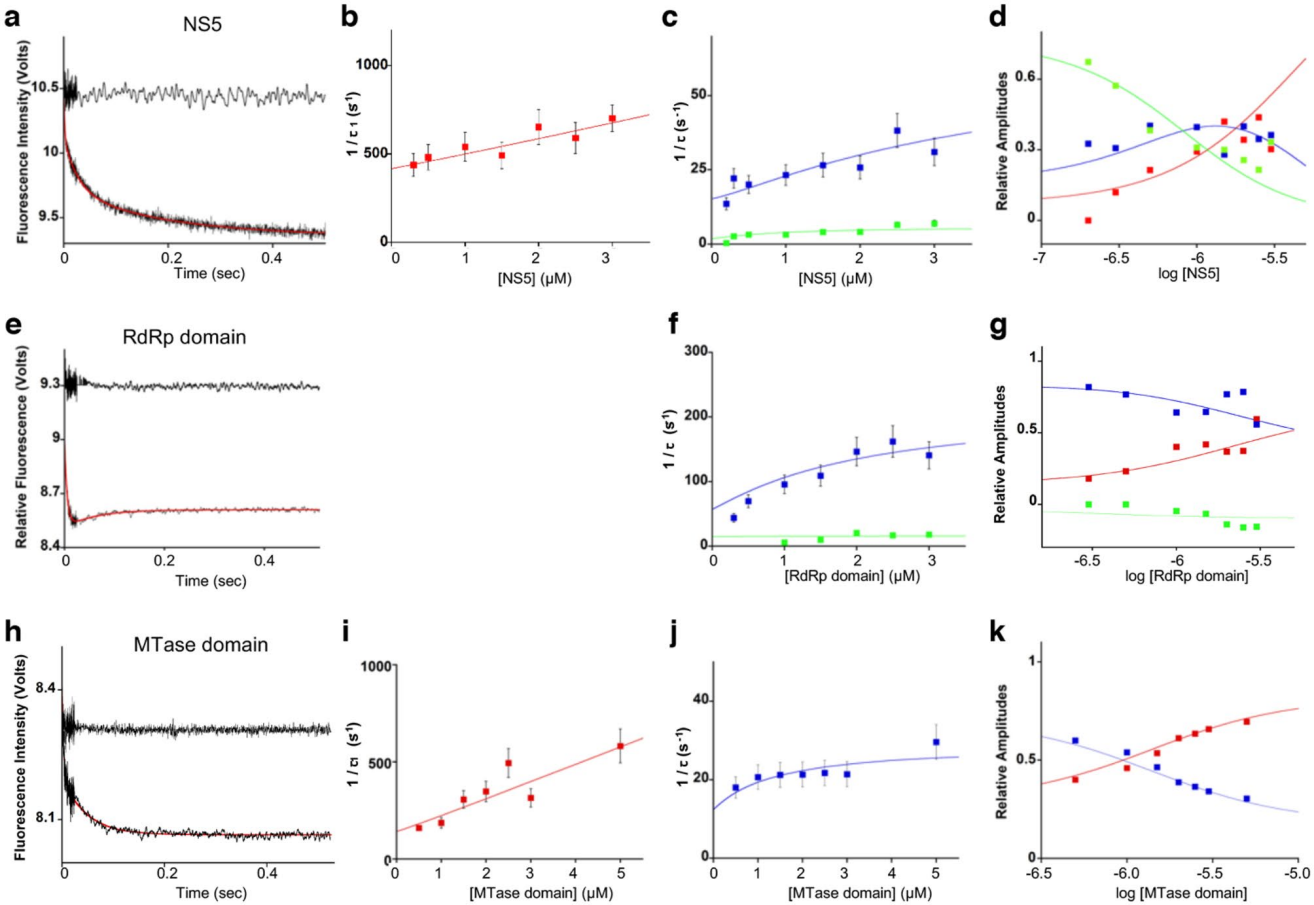
Dynamics of Zika NS5, RdRp and MTase upon SLA binding. (**a**–**d**) The kinetic measurement for ZIKV NS5 and SLA interaction. Fluorescence stopped-flow trace after mixing the fluorescein-labeled ZIKV SLA (SLA-F) with NS5 or buffer (top trace) are shown in (**a**). The solid red line is the nonlinear least-squares fit of the experimental curve using Eq. (12) with a three-step relaxation process. The dependence of the reciprocal relaxation times 1/τ_1_ (red), 1/τ_2_ (blue) and 1/τ_3_ (green) upon the total NS5 concentration are shown in (**b**,**c**). The dependence of the normalized, individual amplitudes of the observed relaxation processes, A_1_ (red), A_2_ (blue) and A_3_ (green) upon the logarithm of the total NS5 concentration are shown in (**d**). The solid lines in (**b**–**d**) are the global fits of the experimental curves with a three-step relaxation process. (**e**–**g**) The kinetic measurement for ZIKV RdRp domain and SLA interaction. A fluorescence stopped-flow traces after mixing Zika SLA-F with the RdRp domain or buffer (top trace) are shown in (**e**). The solid red line is the nonlinear least-squares fit of the experimental curve with a two-step relaxation process (Eq. (12)). A fast step beyond the resolution of the stopped-flow instrument precedes the two observed relaxation steps (see text). The dependence of the reciprocal relaxation times 1/τ_2_ (blue) and 1/τ_3_ (green) upon the total RdRp concentrations are shown in (**f**). The dependence of the normalized relaxation amplitudes A_1_ (red), A_2_ (blue) and A_3_ (green) upon the logarithm of the total RdRp concentrations are shown in (**g**). A_1_ was obtained by subtracting A_2_ and A_3_ from the total amplitude. The solid lines in (**f**,**g**) are global fits of the curves to the three-step sequential mechanism. (**h**–**k**) The kinetic measurement for ZIKV MTase domain and SLA interaction. A fluorescence stopped-flow traces after mixing the Zika SLA-F with the MTase domain or buffer (top trace) are shown in (**h**). The solid red line is the nonlinear least-squares fit of the experimental curve with a two-step relaxation process (Eq. (12)). The dependence of reciprocal relaxation times 1/τ_1_ (red) and 1/τ_2_ (blue) upon the MTase concentration are shown in (**i**,**j**), respectively. The dependence of amplitudes A_1_ (red) and A_2_ (blue) upon the logarithm of the MTase concentrations are shown in (**k**). The solid lines in (**i**–**k**) are the global fits of the curves with a two relaxation-step process.

Next, we examined the kinetic mechanism of ZIKV SLA binding to full-length NS5 and the isolated RdRp and MTase domains by fluorescence stopped-flow methods. All stopped-flow experiments were performed under pseudo-first-order conditions with respect to the protein concentration by mixing SLA-F with a large excess of ZIKV NS5, the RdRp domain or the MTase domain. An example of the stopped-flow kinetic trace of SLA-F fluorescence after mixing with ZIKV NS5 is shown in Fig. 5a. The best fit for the NS5–SLA kinetic traces was obtained using a triple-exponential function, indicating that the simplest mechanism that accounts for the observed change of relaxation times is a minimum three-step binding process. The SLA-F alone showed no fluorescence changes when mixed with the buffer solution without NS5 protein (Fig. 5a, the trace on top). To determine the kinetic mechanism, the stopped-flow experiments were carried out at various NS5 concentrations (0.3–3 µM) with 15 nM of SLA-F. The kinetic traces were then fit using a triple-exponential function to determine the reciprocal relaxation times (λ_i_ or 1/τ_i_, i = 1, 2, 3) and the fractional individual amplitudes (A_i_, i = 1, 2, 3) of the NS5–SLA complex formation with nonlinear least-squares fits (Eq. (12) in “Methods” section). The dependencies of the reciprocal relaxation times, 1/τ_1_, 1/τ_2_, and 1/τ_3_ plotted as a function of the NS5 protein concentrations are shown in Fig. 5b and c. The plot of the reciprocal relaxation time of the first exponential phase, 1/τ_1_*versus* NS5 concentrations showed a linear dependence, indicating a bimolecular association (Fig. 5b). The reciprocal relaxation times 1/τ_2_ and 1/τ_3_ increased in a hyperbolic manner with NS5 concentrations, suggesting intramolecular transitions of the NS5–SLA complex (i.e., conformational changes) in these steps (Fig. 5c). The dependencies of the individual amplitudes, A_1_, A_2_, and A_3_, on the NS5 concentration are shown in Fig. 5d. The individual amplitudes are normalized and expressed as fractions of the total amplitude, A_i_/∑A_i_. At low NS5 concentrations, the total observed amplitude, A_T_, is dominated by A_3_. With increasing protein concentration, contributions of A_1_ and A_2_ increase, with A_1_ becoming dominant at higher protein concentrations.

To globally fit the kinetic data at various NS5 concentrations and obtain rate constants, the stopped-flow kinetic data were also analyzed using the matrix projection operator technique^38,39^. The three-step binding process is described by Eq. (1):

1 $${\text{NS}}5 + {\text{SLA}}\underset{{k_{ - 1} }}{\overset{{k_{1} }}{\rightleftharpoons}}({\text{NS}}5 - {\text{SLA}})_{1} \underset{{k_{ - 2} }}{\overset{{k_{2} }}{\rightleftharpoons}}({\text{NS}}5 - {\text{SLA}})_{2} \underset{{k_{ - 3} }}{\overset{{k_{3} }}{\rightleftharpoons}}({\text{NS}}5 - {\text{SLA}})_{3}$$

The partial equilibrium constants for each step of the above reaction are related to the K_overall_ as,

2 $${\text{K}}_{{{\text{overall}}}} = {\text{ K}}_{1} \left( {1 + {\text{K}}_{2} + {\text{K}}_{2} {\text{K}}_{3} } \right)$$

where K_1_ = k_1_/k_−1_, K_2_ = k_2_/k_−2_, and K_3_ = k_3_/k_−3_. The six kinetic rate constants for the three kinetic steps, as well as fluorescence changes characterizing individual intermediates, are related through the overall binding constants and the maximum fluorescence change ΔF_max_. Using the overall binding constants (K_overall_ = 3.5 × 10^6^ M^−1^) and the maximum fluorescence change (ΔF_max_ = 0.15) that are independently determined (Fig. 1c), partial equilibrium constants and rate constants were determined (Table 2). The rate constants indicate that the formation of the NS5–SLA complex proceeds first by the fast bimolecular association of NS5 and SLA (k_1_ = 9.5 × 10^7^ M^−1^ s^−1^ and k-_1_ = 360 s^−1^) followed by two structural rearrangement steps (k_2_ = 55 s^−1^, k_−2_ = 12 s^−1^ and k_3_ = 4 s^−1^, k_−3_ = 2.5 s^−1^).

**Table 2:** Thermodynamic and kinetic parameters characterizing Zika NS5, the RdRp domain and the MTase domain interactions with Zika SLA.

Parameter^a^	NS5	RdRp domain	MTase domain
K_overall_ (M^−1^)	3.5 (± 0.6) × 10^6^	1.9 (± 0.3) × 10^6^	1.7 (± 0.3) × 10^6^
∆F_max_	0.15	0.09	0.07
K_1_ (M^−1^)	2.64 (± 0.40) × 10^5^	5.0 (± 1.0) × 10^5^	7.2 (± 1.4) × 10^5^
k_1_ (M^−1^ s^−1^)	9.5 (± 1.8) × 10^7^	1.0 (± 0.2) × 10^9^	9.0 (± 1.8) × 10^7^
k_−1_ (s^−1^)	360 ± 60	2000 ± 300	125 ± 25
K_2_	4.6 ± 0.5	2.0 ± 0.5	1.07 ± 0.25
k_2_ (s^−1^)	55 ± 10	140 ± 50	15 ± 3
k_−2_ (s^−1^)	12.0 ± 1.8	70 ± 9	14.0 ± 2.1
K_3_	1.6 ± 0.4	0.07 ± 0.02	
k_3_ (s^−1^)	4.0 ± 0.6	1.0 ± 0.1	
k_−3_ (s^−1^)	2.5 ± 0.4	15.0 ± 2.2	

^a^The K_overall_ and ∆F_max_ values were determined from the independent fluorescence binding experiments.

The stopped-flow kinetic traces of SLA-F fluorescence after mixing with ZIKV RdRp were analogously analyzed. The best fit for the RdRp-SLA kinetic traces were obtained using a double-exponential function (Fig. 5e). However, the binding process is likely more complex because the sum of the amplitudes of the fitted relaxation steps is significantly smaller than the observed total amplitude, i.e., the fluorescence of free SLA-F is significantly higher than the fluorescence of the first observed data point (Fig. 5e). Thus, there is a fast step which is beyond the resolution of the stopped-flow instrument, which precedes the two observed relaxation steps. Therefore, in the case of the RdRp-SLA interaction, the simplest minimum mechanism is a three-step binding process, where initial recognition occurs through very fast bimolecular association of SLA and RdRp.

3 $${\text{RdRp}} + {\text{SLA}}\underset{{k_{ - 1} }}{\overset{{k_{1} }}{\rightleftharpoons}} ({\text{RdRp}} - {\text{SLA}})_{1}\underset{{k_{ - 2} }}{\overset{{k_{2} }}{\rightleftharpoons}} ({\text{RdRp}} - {\text{SLA}})_{2}\underset{{k_{ - 3} }}{\overset{{k_{3} }}{\rightleftharpoons}} ({\text{RdRp}} - {\text{SLA}})_{3}$$

Even though we cannot determine the value of the reciprocal relaxation time for the first fast relaxation process, 1/τ_1_, we can determine its amplitude, A_1_ by Eq. (4), where A_T_ is the experimentally observed total amplitude of the kinetic trace at a given total concentration of the protein^38^.

4 $${\text{A}}_{1} = {\text{A}}_{T} {-}{\text{ A}}_{2} {-}{\text{ A}}_{3}$$

The dependences of the reciprocal relaxation times (1/τ_2_ and 1/τ_3_) and normalized amplitudes, A_1_, A_2_, and A_3_ of three observed relaxation processes on the concentration of the ZIKV RdRp domain are shown in Fig. 5f and g. At low RdRp domain concentrations, the total observed amplitude, A_T_, is dominated by A_2_. With increasing protein concentrations, the contribution of A_1_ to A_T_ increases while A_3_ remains negative throughout the process. The partial equilibrium constants and kinetic rate constants characterizing the intermediates for the three steps are related to the overall binding constant K_overall_ and the maximum fluorescence change ΔF_max_, as described above (Table 2). In the RdRp-SLA complex formation, the initial recognition occurs through a very fast bimolecular association of SLA and RdRp (k_1_ = 1.0 × 10^9^ M^−1^ s^−1^ and k-_1_ = 2000s^−1^), which is followed by two structural rearrangement steps (k_2_ = 140 s^−1^, k_−2_ = 70 s^−1^ and k_3_ = 1.0 s^−1^, k_−3_ = 15.0 s^−1^). Note the initial bimolecular association and dissociation (k_1_ and k_−1_) of RdRp and SLA are 10 times faster than the initial association of NS5 and SLA.

Lastly, a stopped-flow kinetic trace for the MTase domain and SLA interaction is shown in Fig. 5h. The solid red line is a nonlinear least-squares fit of the data using a double-exponential function, indicating that the simplest mechanism that can account for the data is a two-step binding process (Eq. 5).

5 $${\text{MT}} + {\text{SLA}} \underset{{k_{ - 1} }}{\overset{{k_{1} }}{\rightleftharpoons}} ({\text{MT}} - {\text{SLA}})_{1}\underset{{k_{ - 2} }}{\overset{{k_{2} }}{\rightleftharpoons}} ({\text{MT}} - {\text{SLA}})_{2}$$

The dependencies of the reciprocal relaxation time 1/τ_1_ and 1/τ_2_, characterizing the observed kinetic steps as a function of the total MTase domain concentration are shown in Fig. 5i and j, respectively. The plot of the reciprocal relaxation time of the first exponential phase (1/τ_1_) *versus* MTase concentrations showed a linear dependence, indicating a MTase–SLA complex is formed after the bimolecular event (Fig. 5i). The dependence of the normalized amplitudes, A_1_ and A_2_ upon the total concentration of the ZIKV MTase domain is shown in Fig. 5k. The global fit of the kinetic data at various MTase concentrations provides rate constants for the two steps (Table 2). The kinetic constants indicate that a fast bimolecular association of SLA and the MTase domain (k_1_ = 9.0 × 10^7^ M^−1^ s^−1^ and k-_1_ = 125 s^−1^) is followed by only one structural rearrangement step (k_2_ = 15 s^−1^ and k_−2_ = 14 s^−1^) (Table 2).

## Discussion

### The SLA-binding site on NS5 is located in the thumb and MTase domains

Replication of the viral genome is a complex process that involves the viral RNA and polymerase, as well as other viral and cellular proteins. In the case of flaviviruses, the SLA at the 5′ end of viral RNA plays a critical role by functioning as a promoter of the replication process^10–12^. In order to understand how ZIKV NS5 recognizes the SLA promotor for initiation of RNA synthesis, we examined the roles that each of the NS5 domains play in binding to SLA. Both RdRp and MTase domains bind to SLA. Moreover, the SLA-binding affinities for both domains were similar to each other and were  approximately  two-fold lower than the affinity of the full-length NS5 for SLA. This indicates that ZIKV NS5 recognizes SLA using both RdRp and MTase domains. We have thus identified the SLA-binding sites within the RdRp and MTase domains. In the RdRp domain, only the thumb subdomain mutants show reduced SLA binding. Alanine substitutions of R771, R772 and R775, and additional K843 and R844 substitutions in ZIKV NS5 significantly reduced the SLA binding affinity from 280 nM (wild-type NS5) to 500 and 710 nM, respectively (Fig. 3c and Table 1). This suggests that a large positively charged patch consisting of R771, R772, R775, K843 and R844 in the thumb subdomain engages SLA and mediates the formation of the NS5–SLA complex. Interestingly, the single NS5 mutations (R775A and R891A) or a double mutation (K843A-R844A) did not significantly affect the ZIKV NS5–SLA interactions (Table 1), suggesting that the SLA-binding surface on NS5 encompasses a cluster of residues, and other positively charged residues within the patch can compensate for the loss of one or two positively charged residues. The SLA-binding site identified here does not overlap with the template-binding site or the dsRNA exit site in the RdRp domain, suggesting that NS5 has two RNA binding sites, one for ssRNA template, and the other for SLA. Consistent with this idea, wild-type and the SLA-binding site mutant NS5 (R771A-R772A- R775A-K843A-R844A) bind ssRNA with similar affinities (Fig. 3e). In the MTase domain, the mutations of K28E-K29E-R41E-R42E reduced SLA binding affinity from 310 nM (wild-type MTase) to 1.25 µM, suggesting that this four-residue mutation within the MTase domain eliminates specificity for SLA. An NS5 construct containing mutations in both RdRp and MTase patches was expressed, but the protein was insoluble upon *E. coli* expression preventing further investigations. The identified SLA-binding residues in MTase and RdRp domains are highly conserved in flavivirus NS5 (Fig. S2), and residues in the thumb subdomain have previously been shown to be important for viral replication. Individual mutations of R770A and R773A, and a double mutation of K840A-R841A in a DENV2 replicon (corresponding to R772, R775, K843 and R844 in ZIKV NS5) delayed or abolished viral replication^32^. These residues are not directly involved in the catalysis of RNA synthesis, and thus these mutants may abolish viral replication by diminishing interactions with SLA.

The two positively charged patches in the thumb subdomain of RdRp and MTase domains are located on the same surface of NS5, strongly suggesting that the SLA-binding site encompasses both domains. During the viral replication cycle, NS5 interacts with SLA at least twice. First, NS5 must recognize the SLA at the 5′ end of the genome to initiate negative-strand RNA synthesis at the 3′ end (i.e. to carry out the polymerase function of NS5)^10–12^. Second, NS5 must again bind SLA to methylate the cap at the 5′ end of the nascent positive-strand RNA (i.e., to carry out the MTase function of NS5)^29^. These two functionally different NS5 and SLA interactions would engage the same binding site, since it seems unlikely that NS5 possesses two separate SLA-binding sites. In such a case, both MTase and RdRp domains are likely involved in SLA binding. The positively charged patches in RdRp and MTase are separated by ~ 55–60 Å. Based on the secondary structure prediction (Fig. 1a), ZIKV SLA is a long molecule consisting of at least 23 base pairs (~ 60 Å) without counting the bulge between the top and bottom stems or the top loop. Thus, SLA could simultaneously bind both the RdRp and MTase patches.

### Zika NS5 initially recognizes SLA via the RdRp domain

**Figure 6:**
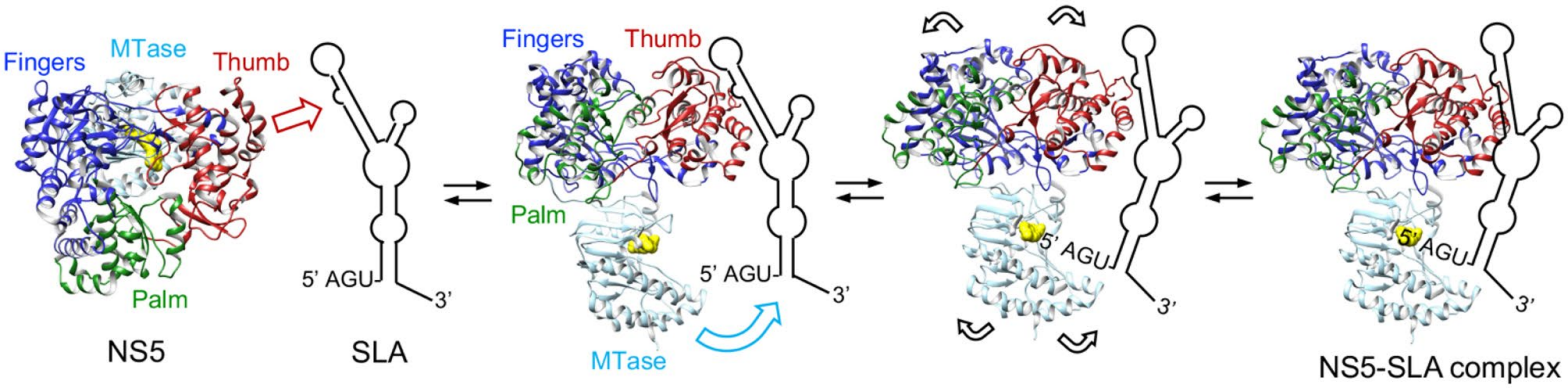
Model of Zika NS5–SLA recognition and complex formation. Zika NS5 recognizes SLA via the thumb subdomain of the RdRp domain. Following the fast binding of the RdRp domain and SLA, two additional conformational changes that involve both RdRp and MTase domains occur. Kinetic measurements suggest that the MTase domain is not involved in the initial SLA recognition but helps to stabilize the NS5–SLA complex. The RdRp and MTase domains are colored as in Fig. 2. The active site in MTase domain is indicated by the bound S-adenosyl homo cysteine (yellow), the byproduct of the MTase reaction. The SLA molecule is oriented based on the requirement of the 5′ cap for MTase activity and previous binding data. The 5′ terminal AGU is required to bind the MTase domain^35^, and thus positioned near the MTase active site. The top stem-loop of SLA is oriented toward the RdRp domain because the top stem-loop is suggested to bind the RdRp domain in the RNase protection assay^10^.

We have examined the dynamics of the NS5–SLA interaction using stopped-flow techniques. The NS5 recognition of SLA occurs through a three-step mechanism. The first step is a fast, bimolecular association of NS5 and SLA, which is followed by two steps in which structural rearrangements of the NS5–SLA complex occur (Fig. 6). Interestingly, the RdRp domain interactions with SLA also occur through a three-step process, but the first step is significantly faster than for the full-length NS5 (k_1−RdRp_ = 1.0 × 10^9^ M^−1^ s^−1^ vs k_1−NS5_ = 9.5 × 10^7^ M^−1^ s^−1^). On the other hand, the RdRp has a large reverse rate constant (k_−1−RdRp_ = 2000s^−1^), indicating that the RdRp-SLA complex dissociates more easily than the NS5–SLA complex where the reverse rate constant is much lower (k_−1−NS5_ = 360 s^−1^). This indicates that the MTase domain in the intact NS5 slows the recognition step but increases the stability of the formed complex. Accordingly, binding of the MTase domain to SLA is characterized by only a two-step mechanism and the initial recognition step is much slower than the RdRp domain (k_1−MTase_ = 9.0 × 10^7^ M^−1^ s^−1^ vs. k_1−RdRp_ = 1.0 × 10^9^ M^−1^ s^−1^). Therefore, NS5 likely recognizes SLA first through the RdRp domain. Taken together, we propose an NS5–SLA interaction model wherein the first fast bimolecular association between NS5 and SLA is carried out via the RdRp domain involving the thumb subdomain (Fig. 6). This fast recognition step is then followed by two steps corresponding to two conformational changes, during which NS5 locks itself onto the SLA through the RdRp and MTase domain to form a stable NS5–SLA complex. Crystal structures of flavivirus NS5s show two different relative arrangements of the RdRp and MTase domains, suggesting that interaction between the RdRp and MTase domains is not fixed spatially^16–20,40^. Thus, it is likely that NS5 adopts several conformations by rearrangement of RdRp and MTase domains to accommodate different RNA molecules such as structured SLA, ssRNA, and dsRNA during viral replication.

## Methods

### Reagents and buffers

A Zika SLA construct containing the first 80 nucleotides with 5′ triphosphate and a 3′ fluorescein tag was obtained from Midland Certified Reagents (Midland, TX). SLA concentration was determined using the extinction coefficient of SLA $\upepsilon_{260}$ = 682,500 cm^−1^ M^−1^. The etheno-modified A(pA)_19_, εA(pεA)_19_ was obtained by modification of the oligomer with chloroacetaldehyde as described previously^31^. The concentration of εA(pεA)_19_ was determined using the extinction coefficient, $\upepsilon_{257}$ = 3,700 cm^−1^ M^−1^ (nucleotide)^31^.

### Expression and purification of Zika NS5

Full-length ZIKV NS5 (GenBank: KY559015.1) and its RdRp domain constructs were obtained from Dr. Cheng Kao (Indiana University)^19^. Full-length ZIKV NS5 and the RdRp domain were expressed with an N-terminal hexahistidine tag and a Small Ubiquitin-like Modifier (SUMO) tag. The gene encoding the MTase domain (GenBank: KU955593.1) was synthesized with an N-terminal hexahistidine tag. All three proteins, the full-length NS5, the RdRp and the MTase domain, were expressed in Rosetta (DE3) *Escherichia coli* cells. Briefly, the cells were grown at 37 °C in Luria Broth (LB) containing 100 μg/ml ampicillin to an OD_600_ of 0.6–0.7. Protein expression was induced by the addition of 1 mM isopropyl β-D-1-thiogalactopyranoside and cell growth continued overnight at 18 °C. For protein purification, the cells were resuspended in lysis buffer containing 50 mM Tris (pH 8.0), 500 mM NaCl, 2 mM β-mercaptoethanol, and 1 tablet of EDTA-free protease inhibitor (Roche Applied Science, Penzberg, Germany), and lysed by sonication. After centrifugation to clear the insoluble debris, the protein in the soluble fraction was purified by Talon metal affinity chromatography (Clontech, Mountain View, CA). NS5 was eluted with a 10–200 mM gradient of imidazole in 50 mM Tris (pH 7.0) buffer containing 500 mM NaCl, 2 mM β-mercaptoethanol and 10% glycerol. The fractions containing NS5 protein were collected and concentrated using an Ultrafree centrifugal filter device (Millipore, Billerica, MA). For the full-length NS5 and RdRp domain, the SUMO and hexahistidine tag was cleaved from the protein by incubation with SUMO protease (1:100 w/w ratio). Subsequently, the cleaved fractions were loaded on a Superdex 200 size exclusion column (GE Healthcare Life Sciences, Marlborough, MA), equilibrated with buffer containing 20 mM Tris–HCl (pH 7.0), 500 mM NaCl, 2 mM β-mercaptoethanol and 20% glycerol. The MTase domain was purified similarly using Talon metal affinity and size-exclusion chromatography.

Mutations within the ZIKV NS5 and the MTase domain were introduced by site-directed mutagenesis using the PfuUltra II Fusion HS DNA Polymerase kit (Agilent Technology, CA). The primers used for mutagenesis are listed in Table S1. Mutations were confirmed by DNA sequencing at the molecular genomics facility at UTMB. Mutant proteins were purified similarly to wild-type proteins. Purified wild-type and mutant proteins were analyzed by SDS-PAGE (Figs.1b, 3a, 4d). Uncropped images of SDS-PAGE gels are shown in Fig. S3.

### Fluorescence measurements

Steady-state fluorescence titrations were performed using an ISS PC1 spectrofluorometer (ISS, Urbana, IL). Polarizers were placed in excitation and emission channels and set at 90° and 55° (magic angle), respectively, in order to avoid possible artifacts due to fluorescence anisotropy of the sample. Experiments were carried out by adding Zika NS5 proteins or the RdRp/MTase domain to a solution containing fluorescein-labeled Zika SLA. A standard buffer B1, containing 50 mM Tris adjusted to pH 8.0 with HCl at 20 °C, 150 mM NaCl, 1 mM MgCl_2_, 2 mM β-mercaptoethanol, and 10% glycerol, was used in fluorescence experiments. Each experiment was carried out in triplicate. The binding of ZIKV NS5 to SLA was monitored by measuring changes in the signal originating from the fluorescein attached to SLA (λ_ex_ = 480 nm, λ_em_ = 520 nm). Titration curves were fit using Kaleida Graph software (Synergy Software, PA). In these experiments, the relative fluorescence change (ΔF_obs_) is defined as (*F*_*i*_*—F*_*0*_*)*/*F*_*0*_, where *F*_*i*_ is the fluorescence of the RNA at a given titration point and *F*_*0*_ is the initial value of the fluorescence of the sample. Both *F*_*0*_ and *F*_*i*_ were corrected for background fluorescence at the applied excitation wavelength. Interactions between the NS5 proteins (wild-type and R771A-R772A-R775A-K843A-R844A) and ssRNA were measured with etheno-labeled εA(pεA)_19_ in buffer B1 (50 mM Tris adjusted to pH 8.0 with HCl at 20 °C, 150 mM NaCl, 1 mM MgCl_2_, 2 mM β-mercaptoethanol, and 10% glycerol). The εA(pεA)_19_ samples were excited at λ_ex_ = 325 nm and emission was recorded at λ_em_ = 410 nm^31^.

Interactions between the MTase domain proteins (wild-type and K28E-K29E-R41E-R42E) and fluorescein-labeled SLA were analyzed using fluorescence anisotropy (r), because fluorescence intensity changes were too small to accurately determine the binding constant for the mutant. Buffer B2, containing 50 mM Tris adjusted to pH 8.0 with HCl at 20 °C, 100 mM NaCl, 1 mM MgCl_2_, 2 mM β-mercaptoethanol, and 10% glycerol, was used for anisotropy measurement. Fluorescence anisotropy (r) is defined as:

6 $$r = \frac{{I_{VV} - GI_{VH} }}{{I_{VV} + 2GI_{VH} }}$$

where I_VV_ and I_VH_ are fluorescence intensities where the first and second subscripts refer to vertical (V) polarization of the excitation and vertical (V) or horizontal (H) polarization of the emitted light. The factor, G = I_HV_/I_HH_, corrects for the different sensitivity of the emission monochromator for vertically and horizontally polarized light. The samples in the fluorescence anisotropy experiments were excited at 480 nm and the emission recorded at 520 nm (fluorescein peaks for excitation and emission, respectively). Each experiment was carried out in triplicate. Interactions between the MTase domain proteins (wild-type and K28E-K29E-R41E-R42E) and etheno-labeled εA(pεA)_19_ were measured in buffer B2 using fluorescence anisotropy. The fluorescence signal of εA(pεA)_19_ were monitored with λ_ex_ = 325 nm and λ_em_ = 410 nm^31^. Each experiment was carried out in triplicate.

### Fluorescence signal analysis

The binding constant, K_1_, characterizing the SLA association with NS5, is defined as

7 $${\text{K}}_{1} = \frac{{[{\text{C}}_{1} ]_{{\text{F }}} }}{{\left[ {{\text{SLA}}]_{{\text{F }}} } \right[{\text{NS}}5]_{{\text{F }}} }}$$

where [C_1_]_F_ is the concentration of the formed complex.

The observed fluorescence of the sample at any point of the titration is defined as

8 $${\text{F}}_{{{\text{obs}}}} = {\text{F}}_{{\text{F}}} \left[ {{\text{SLA}}} \right]_{{\text{F}}} + {\text{F}}_{{\text{C}}} \left[ {{\text{C}}_{1} } \right]_{{\text{F}}}$$

where F_F_ and F_C_ are the molar fluorescence intensities of the free SLA and the formed complex C_1_, respectively. Thus,

9 $${\text{F}}_{{{\text{obs}}}} = {\text{F}}_{{\text{F}}} \left[ {{\text{SLA}}} \right]_{{\text{F}}} + {\text{F}}_{{\text{C}}} {\text{K}}_{1} \left[ {{\text{SLA}}} \right]_{{\text{F}}} \left[ {{\text{NS5}}} \right]_{{\text{F}}}$$

The mass conservation equation for the total SLA concentration, [SLA]_T,_ in the sample is

10 $$\left[ {{\text{SLA}}} \right]_{{\text{T}}} = \left[ {{\text{SLA}}} \right]_{{\text{F}}} + \left[ {{\text{C}}_{1} } \right]_{{\text{F}}} = \, \left[ {{\text{SLA}}} \right]_{{\text{F}}} \left( {1 + {\text{K}}_{1} \left[ {{\text{NS5}}} \right]_{{\text{F}}} } \right)$$

Using Eqs. (9) and (10), the relative observed change of the SLA fluorescence, ∆F_obs_, is then

11 $$\Delta {\text{F}}_{{\text{obs }}} = \frac{{{\text{F}}_{{\text{obs }}} }}{{{\text{F}}_{{{\text{F}} }} [{\text{SLA}}]_{{{\text{T}} }} }} = \frac{1}{{1 + {\text{K}}_{1 } [{\text{NS}}5]_{F } }} +\Delta {\text{F}}_{\max } \left[ {\frac{{{\text{K}}_{1 } [{\text{NS}}5]_{{{\text{F}} }} }}{{1 + {\text{K}}_{1 } [{\text{NS}}5]_{{{\text{F}} }} }} } \right]$$

where ∆F_max_ = F_C_/F_F_, is the maximum value of the observed relative fluorescence quenching. Fluorescence titration curves were fitted using Eq. (11) to determine binding constants. The goodness of the fits were determined using standard chi-square test, which provides the confidence level for the selected binding model of ≥ 0.98.

### Stopped-flow kinetics

All fluorescence stopped-flow kinetic experiments were performed using SX.MV20 stopped-flow instrument (Applied Photophysics Ltd. Leatherhead, UK). Fluorescein-labeled SLA (15 nM) was mixed with 7–8 concentrations of NS5 (300 nM—3 µM), RdRp (300 nM—3 µM) or MTase (500 nM—5 µM) under pseudo-first-order conditions ([protein] >  > [RNA]), and the fluorescence signal upon complex formation was monitored as a function of time with λ_ex_ = 480 nm and λ_em_ = 520 nm. Generally, 10–12 traces were collected and averaged for each concentration of proteins. The kinetic curves were fitted to extract relaxation times and corresponding amplitudes using nonlinear least-squares fitting software provided by the manufacturer, with the exponential function defined as

12 $$F\left( t \right) = F\left( \infty \right) + \mathop \sum \limits_{i = 1}^{n} A_{i}\exp \left( { -\uplambda _{i} {\text{t}}} \right)$$

where F(t) is the fluorescence intensity at time t, F(∞) is the fluorescence intensity at t = ∞, A_i_ is the amplitude corresponding to ith relaxation process, λ_i_ is the time constant (reciprocal relaxation time) characterizing the relaxation process, and n is the number of relaxation processes. Global analyses of the stopped-flow kinetic data were performed using the matrix projection operator technique ^38,39^. All analyses of the data were performed using Mathematica (Wolfram, Urbana, IL) and Kaleida Graph (Synergy Software, PA) as previously described^38,39^.

### Circular dichroism (CD) spectrometry

The far-UV circular-dichroism measurements for wild-type and mutant proteins were performed on a model No. J-815 spectrometer (JASCO, Oklahoma City, OK). The protein concentration was ~ 0.1 mg/ml in 50 mM Tris buffer (pH 7.4) containing 150 mM NaCl, 10% glycerol and 1 mM MgCl_2_. CD spectra were collected at 20 °C in a 0.2-cm path-length cuvette from 195 to 260 nm. Three scans were averaged in the range of 200–250 nm. Data were converted from millidegrees to mean residue ellipticity (MRE) using the formula:

$$\left[ \theta \right] \, = \, (\theta \times 10^{6} )/(c \times l \times n)$$

where [*θ*] is the molar residue ellipticity, *θ* is the CD signal in millidegrees, *c *is the protein concentration in µM, *l *is the path length in mm, and *n *is the number of amino acid residues. The CD data analysis program BeStSel was used to analyze the data and estimate the secondary structure^41^.

## Supplementary information

Supplementary information.
