# Supplementary material for: Identification of the viral RNA promoter stem loop A (SLA)-binding site on Zika virus polymerase NS5
Source: Sci Rep. 2020 Aug 6;10:13306. doi: 10.1038/s41598-020-70094-y (PMC7413259; doi:10.1038/s41598-020-70094-y)
Supplement: Supplementary file 1 — Supplementary information. [file 41598_2020_70094_MOESM1_ESM.docx]

Supplementary Information for

**Identification of the viral RNA promoter stem loop A (SLA)-binding site on**

**Zika virus polymerase NS5**

Paul J. Bujalowski^1^, Wlodzimierz Bujalowski^1^, and Kyung H. Choi ^1, *^

^1^ Department of Biochemistry and Molecular Biology, Sealy Center for Structural Biology and Molecular Biophysics, The University of Texas Medical Branch, Galveston, TX 77555, USA

Table S1. Primers used in mutagenesis reactions

Fig. S1. Zika SLA and MTase domain interaction in buffer B2

Fig. S2. NS5 Sequence alignment between Flaviviruses

Fig. S3. NS5 mutant proteins analyzed by SDS-PAGE

**Table S1.** Primers used in mutagenesis reactions

| Mutations | Forward (F) and reverse (R) primers |
| --- | --- |
| K28E-K29E | F: CCTGGAGTTCTACTCCTACGAGGAGTCAGGCATCACCGAGG  R: ACCTCGGTGATGCCTGACTCCTCGTAGGAGTAGAACTCCAGG |
| R41E-R42E | F: GGTGTGCAGAGAAGAGGCCGAGGAGGCCCTCAAGGACGGTGTGG  R: CCACACCGTCCTTGAGGGCCTCCTCGGCCTCTTCTCTGCACACC |
| K388A-K390A-R391A | F: CCTGGTTGTGGAAAGAGCTAGGCGCACACGCAGCGCCACGAGTCTGTACC  AAAGAAG  R: CTTCTTTGGTACAGACTCGTGGCGCTGCGTGTGCGCCTAGCTCTTTCCAC  AACCAGG |
| R640A-R641A | F: CAGGATCTGTGGCTGCTGGCAGCGTCAGAGAAAGTGACCAACTG  R: CAGTTGGTCACTTTCTCTGACGCTGCCAGCAGCCACAGATCCTG |
| R775A | F: CCGCCGCGACCTGGCCCTGATGGCGAAC  R: GTTCGCCATCAGGGCCAGGTCGCGGCGG |
| R771A-R772A-R775A | F: CTGCTGTATTTCCACGCCGCCGACCTGGCCCTGATGGCGAACGC  R: GCGTTCGCCATCAGGGCCAGGTC GGCGGCGTGGAAATACAGCAG |
| K843A-R844A | F: TGGACGGATATTCCGTATCTGGGTGCAGCTGAAGATCTGTGGTGCG  R: CGCACCACAGATCTTCAGCTGCACCCAGATACGGAATATCCGTCCA |
| R858A | F: CGGTCACCGTCCGGCTACCACCTG GGCA  R: TGCCCAGGTGGTAGCCGGACGGTGACCG |
| R891A | F: CTGTCCACCCAGGTTGCTTACCTGGGCGAAGA  R: TCTTCGCCCAGGTAAGCAACCTGGGTGGACAG |

**
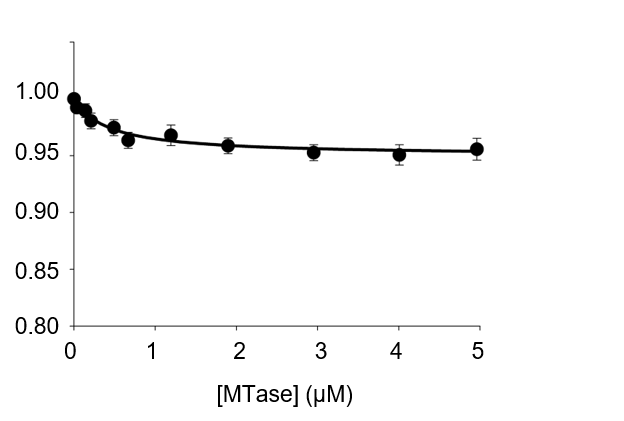
**

**Figure S1. Zika SLA and MTase domain interaction in buffer B2.** Fluorescence titrations of fluorescein-labeled ZIKV SLA with the MTase domain were carried out with 15 nM fluorescein-labeled SLA in buffer B2 (50 mM Tris, pH 8.0, 100 mM NaCl, 1 mM MgCl_2_, 2 mM β-mercaptoethanol and 10% glycerol) at 20 °C with λ_ex_ = 480 nm, λ_em_ = 520 nm. The solid lines are nonlinear least squares fits of the titration curve (equation 6) with K=2.5 x 10^6^ M^-1^. Each experiment was carried out in triplicate. Error bars represent the standard deviations.

Figure S2. Sequence alignment of flavivirus NS5. The SLA-binding site sequences from Zika (ZIKV, GenBank accession code KY559015.1), Dengue (DENV3, GenBank accession code ABV03585.1), Japanese encephalitis (JEV, GenBank accession code M55506.1), and West Nile viruses (WNV, GenBank accession code AF404756.1) are aligned. The identified SLA-binding site residues are colored in red. Overall sequence identity among the four listed flavivirus NS5 is 55%.

**
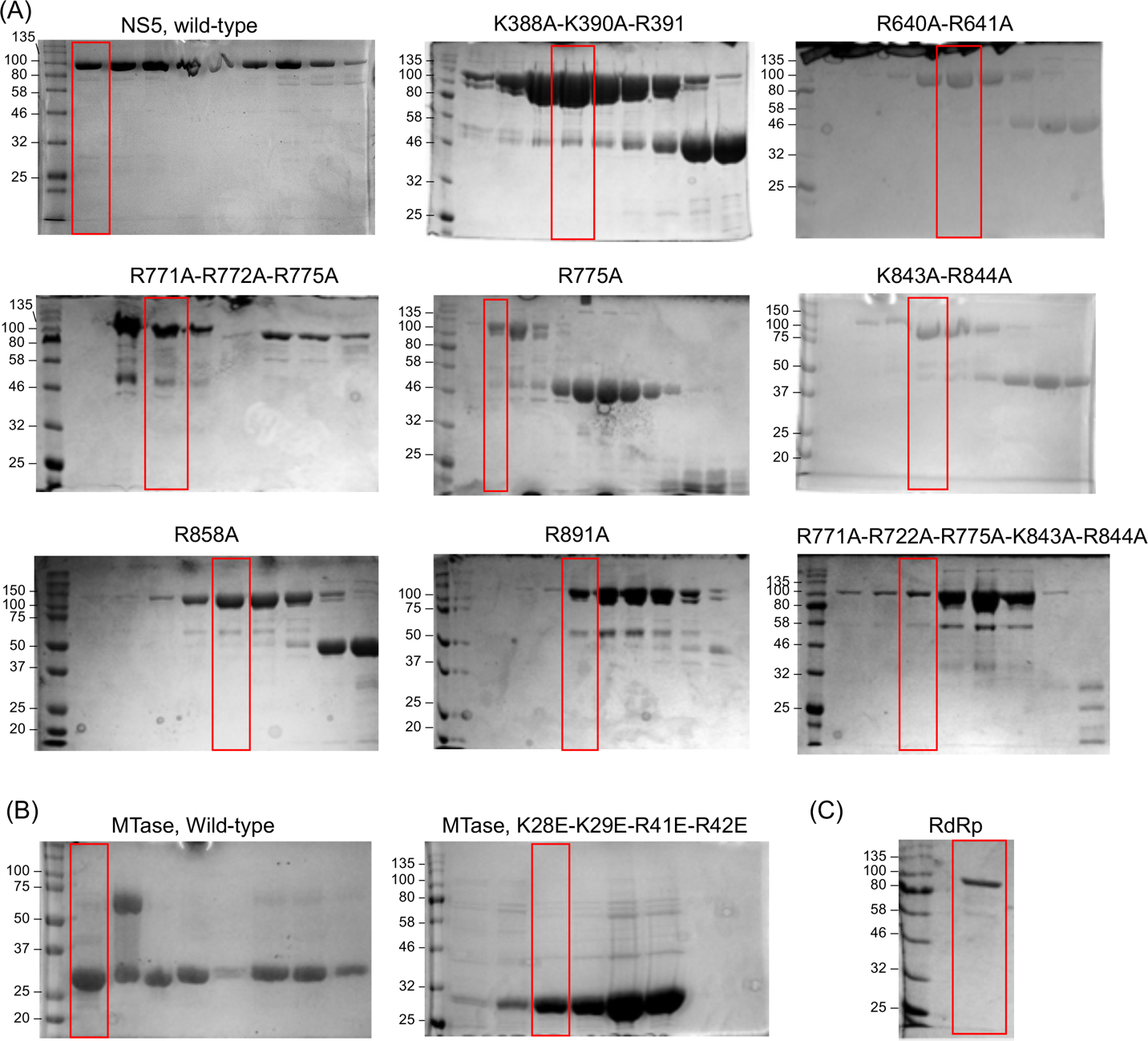
**

**Figure S3. NS5 mutants analyzed by SDS-PAGE. a** SDS-PAGE analysis of wild-type and mutant NS5. Protein fractions from size-exclusion chromatography are shown. The lane with a red box was used in our manuscript. Positions of the molecular weight marker are shown on the left. The full-length NS5 is 100 kDa. **b** SDS-PAGE analysis of wild-type and mutant methyltransferase (MTase) domain. Molecular weight of MTase is 30 kDa. **c** SDS-PAGE analysis of RNA-dependent RNA polymerase (RdRp) domain. Molecular weight of RdRp is 70 kDa.
